# Untangling the link between experiential avoidance and non-suicidal self-injury: a multidimensional approach

**DOI:** 10.1080/00049530.2024.2315951

**Published:** 2024-02-18

**Authors:** Sophie B Haywood, Penelope Hasking, Mark E Boyes

**Affiliations:** a School of Population Health, Faculty of Health Sciences, Curtin University, Perth, Australia; b Curtin enAble Institute, Faculty of Health Sciences, Curtin University, Perth, Australia

**Keywords:** Experiential avoidance, self-injury, unidimensional, multidimensional, NSSI

## Abstract

**Objective:**

Experiential avoidance, an individual’s unwillingness to experience uncomfortable internal feelings/emotions, has been found to be associated with history of self-injury. This association is mainly found in studies that use global measures of experiential avoidance. However, experiential avoidance is purported to be a multidimensional construct. This study aims to test both unidimensional and multidimensional measures of experiential avoidance and their associations with self-injury.

**Method:**

University students (*n* = 632, *M* = 25.01, *SD* = 7.13, 78.8% female, 70.9% with lived experience of self-injury) completed well-validated self-report measures of self-injury, experiential avoidance (The Brief and the Multidimensional Experiential Avoidance Questionnaire).

**Results:**

As expected, all sub-scales of multidimensional measure of experiential avoidance were highly correlated with the global score for experiential avoidance. The global measure of experiential avoidance differentiated individuals with no history, with past history, and recent history of self-injury. When assessed using the multidimensional measure, only the sub-factors behavioural avoidance and repression and denial differentiated those with no history of self-injury from those with recent history and those with recent history from those with past history of self-injury.

**Conclusion:**

Findings raise the possibility that associations between experiential avoidance and self-injury may be down to two specific aspects of experiential avoidance, namely 1) behavioural avoidance and 2) repression/denial. If true, this will have important theoretical, clinical, and measurement implications for research into self-injury.

## Key Points

**What is already known about this topic**:

1. Self-injury is prevalent among university students.
2. Experiential avoidance is associated with non-suicidal self-injury.
3. Experiential avoidance is a multi-dimensional construct.

**What this topic adds**:

1. Despite the knowledge that experiential avoidance is a multidimensional construct, most studies utilise unidimensional measures to capture the construct.
2. This study explores experiential avoidance from a unidimensional and multidimensional approach.
3. Experiential avoidance is associated with non-suicidal self-injury, however, when we look at the specific aspects of experiential avoidance, only behavioural avoidance and repression/denial retain the association.
4. Understanding the specific aspects of experiential avoidance that are associated with engagement in self-injury can assist in providing more targeted clinical interventions.

Non-suicidal self-injury is the intentional and purposive damage to one’s own body tissue without suicidal intent (International Society for the Study of Self-injury, 2022). Self-injury is pervasive across different age groups, with 17% of adolescents, 13% of young adults, and 5% of older adults reporting a history of self-injury (Swannell et al., 2014). This behaviour is especially common within university students, with 20% reporting a history of self-injury compared to 11% of their peers (Swannell et al., 2014). Additionally, 10.3% of students report onset of engagement in self-injury in their first year of university, with 6% reporting beginning in their second year of university (Kiekens et al., 2019). University students who engage in self-injury are also reported to have poorer mental health outcomes, higher rates of academic failure (Kiekens et al., 2016), encounter increased stigma (Burke et al., 2019), and be at greater risk of attempting suicide (Whitlock et al., 2013). Common methods of self-injury include, among other methods, cutting, scratching, and burning oneself (Klonsky & Muehlenkamp, 2007). The motivations for engaging in self-injury are diverse and multifaceted, including anti-disassociation, self-punishment, and most prominently, emotion regulation (Taylor et al., 2018).

Given that affect regulation is the most endorsed function of self-injury (Taylor et al., 2018), most models of NSSI focus on emotional experience and regulation of that experience (Chapman et al., 2006; Hasking et al., 2017; Nock, 2009; Nock & Prinstein, 2004; Selby & Joiner, 2009). Across these models, the experience and regulation of these emotions play an important role in whether someone is likely to start or continue to engage in self-injury. One such model is the experiential avoidance model of self-injury (Chapman et al., 2006). Experiential avoidance is an individual’s unwillingness to or inability to sit with uncomfortable internal experiences such as emotions, feelings, and thoughts (Hayes et al., 1999). According to the Experiential Avoidance Model of Self-injury, all individuals vary in the extent to which they want to avoid these uncomfortable internal experiences (Chapman et al., 2006). The model outlines a sequence of events wherein the individual encounters a stimulus that elicits an internal experience, such as a thought, feeling, and/or emotion. Individuals with a greater propensity towards the avoidance of these internal experiences are more likely to engage in self-injury to distract from the experience (Chapman et al., 2006).

A recent meta-analysis examined the associations between experiential avoidance and self-injury (Haywood et al., 2023), finding a small-to-medium pooled effect. A consideration raised in the meta-analysis was that all the studies that reported significant associations between experiential avoidance and self-injury used measures that were unidimensional (Haywood et al., 2023). Experiential avoidance has been conceptualised as a multidimensional construct consisting of behavioural avoidance, distress aversion, procrastination, distraction and suppression, repression and denial, and distress endurance (Chawla & Ostafin, 2007; Gámez et al., 2011). However, only two studies (out of 19) used a multidimensional measure. Unfortunately, they did not report on the information in a way that allowed for inclusion in the meta-analysis. Nonetheless, Bentley et al. (2015) found a significant association between the procrastination subscale of the Multidimensional Experiential Avoidance Questionnaire (Gámez et al., 2011) and severity of NSSI. However, Nielsen et al. (2017) did not find any significant associations when using the same measure. These studies highlight the mixed findings regarding specific aspects of experiential avoidance that may be associated with self-injury. It may be important to consider that unidimensional measures could miss the unique aspects of experiential avoidance that are associated with self-injury. Understanding which specific aspects of experiential avoidance are associated with the onset and maintenance of self-injury may have important implications for both the theoretical understanding of the behaviour and interventions.

Given the prevalence of self-injury within university students, the aim of this study was to test both a unidimensional and multidimensional measure of experiential avoidance and their associations with self-injury within this population. Based on previous research, we expect there will be a significant association between experiential avoidance and self-injury when assessed using a unidimensional measure of experiential avoidance. Furthermore, when assessing this relationship using a multidimensional measure of experiential avoidance, we expect that only specific dimensions of experiential avoidance will be associated with self-injury. However, given the exploratory nature of using the multidimensional measure of experiential avoidance, we are unsure of the specific dimensions that will be associated with self-injury.

## Methods

### Participants

Participants were Australian university students (*N* = 632) aged between 19 and 62 years (*M* = 25.01, *SD* = 7.13); 498 identified as women (78.8%); 90 identified as men (14.2%), and 44 self-described (7%; 3 agender, 2 genderfluid/queer, 30 non-binary, 6 trans male, 2 did not specify gender). All participants were enrolled at Australian universities. Information was collected on age, gender, country of birth, and any mental health conditions, including the specific diagnosis.

### Measures

#### Non-suicidal self-injury

Information on NSSI was collected using Section 1 of the inventory of statements about Self-injury (ISAS; Klonsky & Glenn, 2009). Participants were provided with a definition of self-injury and were then asked if they had ever engaged in self-injury. Those who indicated they had engaged in self-injury were asked about how many times they had engaged in the last year, main forms of self-injury, and the age at which they had first engaged in self-injury. The ISAS has good short-term test–retest reliability (1–4 weeks; *r* = .85; Glenn & Klonsky, 2011).

### Experiential avoidance

Experiential avoidance was captured using both a multidimensional and unidimensional measure. The Brief Experiential Avoidance Questionnaire (BEAQ; Gámez et al., 2014) is the short form of the Multidimensional Experiential Avoidance Questionnaire (Gámez et al., 2011). It is a 15 item, unidimensional scale. Participants respond to statements (e.g., *“I go out of my way to avoid uncomfortable situations”*) on a 6-point Likert scale ranging from 1 (strongly disagree) to 6 (strongly agree). Scores range from 15 to 90, with higher scores indicating higher levels of experiential avoidance. The scale has good internal consistency (α =. 86) and good convergent validity with the MEAQ (mean *r* = .62; Gámez et al., 2014). In the current sample, the internal consistency was good (α = .87; ω = .87).

The Multidimensional Experiential Avoidance Model (MEAQ; Gámez et al., 2011) is a 62-item measure that captures various types of experiential avoidance. The subscales include behavioural avoidance (e.g., “*I won’t do something if I think it will make me uncomfortable*”), distress aversion (e.g., *“If I could magically remove all of my painful memories, I would”*), repression/denial (e.g., *“I sometimes have difficulty identifying how I feel”*), distraction/suppression (e.g., *“When something upsetting comes up, I try very hard to stop thinking about it”*), procrastination (e.g., *“I tend to put off unpleasant things that need to get done*”), and distress endurance (e.g., *”People should face their fears”*). Participants rated statements on a 6-point Likert scale ranging from 1 (strongly disagree) to 6 (strongly agree). The measure can be scored as a total score or subscale scores. For this study, the subscale scores were used. Scores for the subscales range from 11 to 66 for behavioural avoidance and distress endurance, 13 to 78 for distress aversion and repression/denial, and 7 to 42 for procrastination and distraction/suppression. Higher scores indicate higher levels of that construct. Internal consistency has been reported as adequate to good across community, student, and clinical (in-patient) samples (α = .76–.95; Gámez et al., 2011). In the current study, the internal consistency for the subscales was good (α = .86–.89; ω = .86–.89).

### Procedure

Following ethics approval from the University Human Research Ethics Committee (HREC2020–0649), the study was advertised and made available on the University’s online research participation pool, as well as being promoted on various social media platforms. Students recruited through the participation pool were awarded course credits. Students who completed the study online were not compensated for their time. Participants were provided with a link to the online survey that stated the objectives of the project, how their data would be stored, confidentiality, and the nature of the survey. Surveys could be completed in participants’ own time. Surveys took approximately 30 min to complete. Once the survey was completed, all participants were provided with a list of useful resources that included information relating to self-injury and counselling services. Data was collected between August 2021 and October 2022.

### Data analysis

Participants were categorised into three groups based on their NSSI history: no history of self-injury; history of self-injury, but not in the last 12 months; and history of self-injury within the last 12 months. Correlations were conducted between all subscales on the Multidimensional Experiential Avoidance Questionnaire and the overall score of the Brief Experiential Avoidance Questionnaire. Multinominal logistic regression was used to assess the overall and unique contributions of unidimensional and multidimensional facets of experiential avoidance on history of self-injury.

## Results

### Preliminary results

All analyses were conducted in SPSS version 28. Two cases had more than 50% of data missing, so they were removed from the dataset. Remaining missing data (≤1.3% across variables) were missing completely at random, χ^2^(3413) = 3453.004. *p* = .312. Expectation maximisation was used to impute the missing data (Tabachnick & Fidell, 2013).

Most participants were born in Australia (*n* = 483, 76.4%), 448 (70.9%) reported a lifetime history of self-injury, and 354 (56%) reported a diagnosis of a mental illness. The most commonly reported diagnoses were comorbid anxiety and depression (54%), anxiety disorder (20%), and depression (13.5%). Of the participants with a history of self-injury, 281 (44.5%) reported engaging in the behaviour in the last year. Age of onset of self-injury ranged from 10 to 36 years (*M* = 13.32, *SD* = 3.79). Most common methods of self-injury included cutting (36.6%), banging or hitting yourself (9.5%), and severe scratching (6.3%). More females (71.9%) than males (51.1%) and all participants who self-reported their gender reported a history of self-injury, χ^2^(2) = 35.37, *p* < .001, Ѵ = .24. Younger participants reported higher levels of experiential avoidance across all subscales of the Multidimensional and Brief Experiential Avoidance Questionnaire (see Table 1). Therefore, age and gender were statistically controlled in the multinominal regression. Large, positive, correlations (*r* > .80) were observed between the total score of the Brief Experiential Avoidance Questionnaire and the behavioural avoidance and distress aversion subscales of the Multidimensional Experiential Avoidance Questionnaire.

**Table 1.** Correlations between variables in the model.

		Never N = 184		Previous History N = 167		Recent History N = 281								
		*M*	*SD*	*M*	*SD*	*M*	*SD*	2	3	4	5	6	7	8
1	Age	25.18	8.28	25.01	6.27	24.90	6.86	−.15***	−.17***	−.17***	−.11**	−.14***	.11**	−.17***
2	Behavioural Avoidance	37.98	9.39	40.22	9.40	39.82	10.15	-	.60***	.54***	.46***	.38***	−.44***	.81***
3	Distress Aversion	45.24	11.62	47.70	11.80	49.40	12.68		-	.42***	.50***	.44***	−.24***	.80***
4	Procrastination	27.59	6.78	29.75	7.07	30.74	7.26			-	.30***	.46***	−.44***	.68***
5	Distraction and Suppression	27.28	6.35	29.16	7.35	28.83	6.32				-	.36***	−.04	.60***
6	Repression and Denial	37.10	11.27	40.56	11.63	44.56	12.15					-	−.17***	.68***
7	Distress Endurance	46.61	7.24	44.85	8.35	43.61	8.78						-	−.42***
8	BEAQ	49.73	12.17	54.13	12.06	54.55	12.01							-

*p < .05. **p < .01. ***p < .001.

### Multinominal logistic regression

#### Unidimensional experiential avoidance and non-suicidal self-injury

A multinominal logistic regression, with the total score for the Brief Experiential Avoidance Questionnaire and controlling for age and gender, significantly differentiated participants with no history of self-injury from those who had previous history of self-injury but not in the last 12 months, and from participants who had self-injured in the last 12 months, χ^2^(6) = 57.053, *p* < .001, Cox and Snell *R*^*2*^ = .10, Nagelkerke *R*^*2*^ = .11. Experiential avoidance was significantly associated with previous and recent engagement in NSSI (see Table 2). A second multinominal logistic regression was conducted with recent history of NSSI as the reference category. Experiential avoidance differentiated participants who had never engaged in NSSI and those with a recent history of the behaviour. No significant differences were observed between those with a past and recent history of NSSI (see Table 2).

**Table 2.** Predictor coefficients for the model predicting history of NSSI using unidimensional measure.

Regression Variable	Past History of NSSI^a^			Recent History of NSSI^a^			Past History of NSSI^b^		
	*B (SE)*	Sig.	Exp (*B*) [95% *CI*]	*B (SE)*	Sig.	Exp (*B*) [95% *CI*]	*B (SE)*	Sig.	Exp (*B*) [95% *CI*]
Intercept	−3.23 (.86)	***p* < .001**		−4.23 (.80)	***p* < .001**		1.00 (.80)	*p* = .213	
BEAQ	.03 (.01)	***p* = .006**	1.03 [1.01, 1.05]	.04 (.01)	***p* < .001**	1.05 [1.03, 1.06]	−.02 (.01)	*p* = .053	.98 [.97, 1.00]
Age	.00 (.02)	*p* = .818	1.00 [.97, 1.04]	.01 (.02)	*p* = .683	1.01 [.98, 1.04]	−.00 (.02)	*p* = .998	1.00 [.97, 1.03]
Gender	.89 (.26)	***p* < .001**	2.42 [1.45, 4.05]	1.18 (.24)	**p < .001**	3.27 [2.03, 5.26]	−.30 (.24)	*p* = .742	.74 [.46, 1.19]

^a^Reference category: Never Engaged.

^b^Reference category: Recently Engaged.

#### Multidimensional experiential avoidance and Non-suicidal self-injury

A multinominal logistic regression, with all variables entered simultaneously (controlling for age and gender) and never engaged in self-injury as the reference category, significantly differentiated those with a recent history of self-injury from those with no history and prior history of self-injury, χ^2^(16) = 84.15, *p* < .001, Cox and Snell *R*^*2*^ = .14, Nagelkerke *R*^*2*^ = .16. The subscales of behavioural avoidance and repression and denial significantly differentiated participants who had never self-injured and those with recent engagement in self-injury was significantly associated with a previous and recent history of engagement in self-injury (see Table 3). A second multinominal regression was conducted with recent history of NSSI as the reference category. The subscales of behavioural avoidance and repression/denial differentiated those with a recent and previous history self-injury (see Table 3).

**Table 3.** Predictor coefficients for the model predicting history of NSSI using multidimensional measure.

Regression Variable	Past History of NSSI^a^			Recent History of NSSI^a^			Past History of NSSI^b^		
	*B (SE)*	Sig.	Exp (*B*) [95% *CI*]	*B (SE)*	Sig.	Exp (*B*) [95% *CI*]	*B (SE)*	Sig.	Exp (*B*) [95% *CI*]
Intercept	−3.42(1.41)	***p* = .015**		−2.69 (1.27)	***p* = .035**		−.73 (1.27)	*p* = .564	
Age	.01 (.02)	*p* = .773	1.01 [.97, 1.04]	.01 (.02)	*p* = .519	1.01 [.98, 1.04]	−.01 (.02)	*p* = .740	1.00 [.97, 103]
Gender	.89 (.27)	***p* < .001**	2.43 [1.44,4.12]	1.15 (.25)	*p***< .001**	3.15 [1.94, 5.12]	−.26 (.25)	*p* = .297	.77 [.48, 1.25]
Behavioural Avoidance	.01 (.02)	*p* = .877	1.01 [.97, 1.04]	−.03 (.02)	***p* = .039**	.97 [.94, 1.00]	.04 (.02)*	***p* = .021**	1.04 [1.01, 1.07]
Distress Aversion	.00 (.01)	*p* = .967	1.00 [.98, 1.03]	.02 (.01)	*p* = .165	1.02 [.99, 1.04]	−.02 (.01)	*p* = .173	.98 [.96, 1.01]
Procrastination	.02 (.02)	*p* = .314	1.02 [.98, 1.07]	.03 (.02)	*p* = .161	1.03 [1.00, 1.07]	−.01 (.02)	*p* = .752	.99 [.96, 1.03]
Distraction & Suppression	.02 (.02)	*p* = .459	1.02 [.97, 1.06]	−.01 (.02)	*p* = .639	.99 [.95, 1.03]	.03 (.02)	*p* = .192	1.03 [.99, 1.07]
Repression & Denial	.01 (.01)	*p* = .292	1.01 [.99, 1.04]	.05 (.01)	***p* < .001**	1.05 [1.03, 1.07]	−.04 (.01)	***p* < .001**	.97 [.95, .99]
Distress Endurance	−.00 (.02)	*p* = .872	1.00 [.96, 1.03]	−.03 (.02)	*p* = .094	.97 [.94, 1.01]	.02 (.02)	*p* = .125	1.02 [.99, 1.06]

^a^Reference category: Never Engaged.

^b^Reference category: Recently Engaged.

## Discussion

The aim of this study was to explore the association between experiential avoidance and self-injury using both unidimensional and multidimensional measures of experiential avoidance. Overall, the unidimensional questionnaire differentiated individuals with no history of self-injury from those with a history but who had not engaged in the last 12 months and those with a recent history (had engaged in the last 12 months). However, when analysed using the multidimensional subscales only behavioural avoidance (which was highly correlated with the Brief Experiential Avoidance total score) and repression/denial subscales differentiated those who had a recent history of engagement from those who had no history of engagement and those who had a previous history of engagement. No subscales differentiated those with no history and a previous history of engagement. This may potentially be as a result of the time elapsed for individuals who have a history of self-injury but no longer engage in self-injury. Alternatively, it may be that an individual with self-injury only engaged in the behaviour once and did not find it an effective tool for regulating their emotions. Further work is required to capture the nuances of those who report a history of self-injury but no longer engage in the behaviour.

As expected, given that the Brief Experiential Avoidance Questionnaire (Gámez et al., 2014) is a shortened version of the Multidimensional Experiential Avoidance Questionnaire (Gámez et al., 2011), moderate to large correlations were found between measures. There were large correlations between the behavioural avoidance and distress aversion subscales of the Multidimensional Experiential Avoidance Questionnaire and the total score of the Brief Experiential Avoidance Questionnaire, suggesting these could be responsible for the majority of the associations observed when using the Brief Experiential Avoidance Questionnaire.

Items that load on to the behavioural avoidance subscale of the Multidimensional Experiential Avoidance Questionnaire capture an individual’s tendency to actively avoid situations that they find uncomfortable or physically distressing (e.g., “*I go out of my way to avoid uncomfortable situations*”; Gámez et al., 2011). When we consider the early definition of experiential avoidance being the avoidance of uncomfortable *internal* experiences, this subscale does not appear to be tapping into the construct of experiential avoidance. The Experiential Avoidance Model (Chapman et al., 2006) suggests that a stimulus occurs that elicits an emotional response. However, if individuals are avoiding the situations that evoke the internal response, it suggests that they would not have the resulting uncomfortable internal experiences. The repression and denial subscale of the Multidimensional Experiential Avoidance Questionnaire taps into an individual’s attempt to mentally distance themselves from distressing experiences or feelings or a lack of awareness of one’s feelings or distress (Gámez et al., 2011). However, if individuals attempt to repress or deny an emotion that they consider to be unpleasant, the emotion may actually intensify rather than subside (Amstadter, 2008). While there is no evidence of this specifically in relation to self-injury, given that repressing or denying an emotion may intensify that emotional experience, it is plausible that such a strategy may increase risk of self-injury. However, this would need to be tested in future research.

Together, these findings suggest that further refinement of our existing theoretical understanding of experiential avoidance and self-injury may be required. Our existing models tend to explore avoidance as a global construct (Chapman et al., 2006; Hasking et al., 2017; Nock & Prinstein, 2004; Selby & Joiner, 2009). Within these models, the role of avoidance is purported to play different roles such as the avoidance of unpleasant things or situations (Nock & Prinstein, 2004), internal experiences (Chapman et al., 2006), emotional cascades (Selby & Joiner, 2009), as well as situations and emotions (Hasking et al., 2017). However, the current findings highlight that it may be specific aspects of experiential avoidance that are responsible for this association with self-injury. Additionally, while behavioural avoidance, changing our behaviour to avoid situations, people, or objects that lead to these uncomfortable internal experiences is part of Hayes et al. (1999) description of experiential avoidance, the Experiential Avoidance Model is more focused on avoidance of internal states (Chapman et al., 2006). By refining our models to examine the specific facets of avoidance or experiential avoidance that are associated with the onset and maintenance of self-injury, we will improve our understanding of who is likely to engage in self-injury. More specific models will in turn improve our ability to provide more targeted interventions in clinical settings so that our interventions are focused on the specific facets of avoidance that are associated with why people may engage in self-injury.

### Limitations

When considering the findings of the current study, it is important to do so with some limitations in mind. Firstly, due to the data being cross-sectional, we are unable to draw conclusions regarding the temporal sequencing of events. Secondly, as the survey was advertised as a study specifically exploring self-injury and participants self-selected to take part, the generalisability of the study may be limited. Future research should consider replicating this study within clinical and other community samples. Given the prevalence of self-injury with in clinical inpatients (40–80% of adolescents and 18–20% of adults reporting a lifetime history of self-injury; Briere & Gil, 1998; Darche, 1990; DiClemente et al., 1991; Glenn & Klonsky, 2013; Nock & Prinstein, 2004; Polanco-Roman et al., 2014) and the lower rates within community samples exploring the role of experiential avoidance in self-injury within these populations could enhance our understanding of the association. We also know that experiential avoidance plays a transdiagnostic role in psychopathology within both clinical and community samples (Akbari et al., 2022).

## Conclusion

Non-suicidal self-injury is a prevalent and widespread behaviour associated with adverse consequences, including a greater likelihood of future suicidal ideation and behaviours (Kiekens et al., 2018). It is therefore critical that we have a deeper understanding of the mechanisms associated with the onset and maintenance of self-injury. The findings of the current study suggest that conceptualising experiential avoidance as a global construct may be missing the specific facets of avoidance, such as behavioural avoidance and repression and denial that are involved in why people engage in self-injury. In addition, taking this more fine-grained view highlights that aspects of experiential avoidance (as measured by the Multidimensional Experiential Avoidance Questionnaire) do not map very closely on to experiential avoidance as defined in the Experiential Avoidance Model. Refining our existing theoretical models to only focus on specific aspects of avoidance associated with self-injury, behavioural avoidance, and repression and denial may improve and advance our understanding of who may engage in self-injury. Additionally, by conducting qualitative studies with people who have a history of self-injury, we could gain a deeper understanding of the role of experiential avoidance in self-injury. This in turn can improve clinical interventions to support individuals who engage in self-injury. If other studies replicate these findings, it will have significant conceptual, methodological, and theoretical implications for our existing understanding of the role experiential avoidance plays in self-injury.

## Data Availability

The data that support the findings of this study are available from the corresponding author, [MB], upon reasonable request.
